# Small for gestational age is a risk factor for thyroid dysfunction in preterm newborns

**DOI:** 10.1186/s12887-020-02089-7

**Published:** 2020-04-23

**Authors:** Chunhua Liu, Kaiyan Wang, Jizhong Guo, Jiru Chen, Mei Chen, Zhexi Xie, Pu Chen, Beiyan Wu, Niyang Lin

**Affiliations:** 1 Neonatal Intensive Care Unit, Department of Pediatrics, The First Affiliated Hospital of Shantou University Medical College, 57 Changping Road, Shantou, 515041 Guangdong People’s Republic of China; 2 Medical Informatics Research Center, Shantou University Medical College, 22 Xinlin Road, Shantou, 515041 Guangdong People’s Republic of China

**Keywords:** Thyroid hormone, Thyroid-stimulating hormone, Small for gestational age, Preterm, Newborn, Thyroid dysfunction, Thyroid function test

## Abstract

**Background:**

Thyroid hormones play an important role in the normal growth and maturation of the central nervous system. However, few publications addressed the altered thyroid hormone levels in preterm small for gestational age (SGA) newborns. We hypothesized preterm SGA infants have higher thyroid-stimulating hormone (TSH) concentrations than appropriate for gestational age (AGA) ones within the normal range and an increased incidence of thyroid dysfunction.

**Methods:**

The study was designed to compare thyroid hormone levels within the normal range and the incidence of thyroid dysfunction in the SGA and AGA groups to test the hypothesis. The medical records of all preterm infants admitted to the neonatal intensive care unit (NICU) at the First Affiliated Hospital of Shantou University Medical College, Shantou, China, between January 1, 2015 and December 31, 2018, were reviewed. Blood samples were collected between 72 and 96 h of life and analyzed with TSH, free thyroxine (FT4) and free triiodothyronine (FT3) assays. Thyroid function test (TFT) results, and neonatal demographic and clinical factors were analyzed to identify the associations between SGA birth and altered thyroid concentrations and thyroid dysfunction.

**Results:**

TSH and FT4 concentrations were significantly higher in the SGA group than the AGA group ((3.74(interquartile range (IQR):2.28 ~ 6.18) vs. 3.01(IQR: 1.81 ~ 5.41) mU/L, *p* = 0.018), and (17.76 ± 3.94 vs. 17.42 ± 3.71 pmol/L, *p* = 0.371), respectively). The higher TSH levels were associated with being SGA or Z-score of birth weight (BW) for GA after adjusting for potential confounders ((*β*_*SGA*_ = 0.68 (95% confidence interval (CI) 0.15 ~ 1.21), *p* = 0.013) or (*β*_*Z-score*_ = − 0.25 (95%CI -0.48 ~ − 0.03), *p* = 0.028), respectively). However, we did not find a significant association between SGA birth and altered FT4 concentrations. Furthermore, compared with the AGA group, the SGA group presented an increased incidence of transient hypothyroxinemia with delayed TSH elevation (dTSHe), a higher percentage receiving levothyroxine (L-T4) therapy, and a higher rate of follow-up within the first 6 months of life.

**Conclusions:**

Preterm SGA newborns had significantly higher TSH concentrations within the normal range and an increased incidence of thyroid dysfunction. The SGA newborns with these features should be closely followed up with periodical TFTs and endocrinologic evaluation.

## Background

Being small for gestational age (SGA) is associated with a variety of adverse outcomes, including the impaired performance of cognitive and sensorimotor functions. According to a recent report, most SGA births occur in countries of low and middle income and are concentrated in South Asia, underlining effective interventions to reduce disability, stunting, and non-communicable diseases [1].

Thyroid hormones play an important role in the normal growth and maturation of the central nervous system. Even transient hypothyroxinemia in the first few weeks of life may cause neurologic and mental problems later in life [2]. With the advent of newborn screening (NBS) for congenital hypothyroidism (CH), L-T4 replacement therapy started within 2 weeks of age can normalize thyroxine (T4) and TSH to prevent the developmental deficits resulting from late diagnosis [3].

Several studies suggest that the hypothalamic-pituitary-adrenal axis and thyroid function may regulate pre- and postnatal growth in children born SGA, at least in early life [4, 5]. A recent report revealed TSH concentrations are significantly higher in preterm SGA newborns, suggesting the elevation should be taken into consideration when establishing a reference interval for this population [6].

Furthermore, most SGA infants will experience catch up growth (CUG) during early childhood, and the patterns of CUG are affected by hypothyroidism and following L-T4 replacement therapy [7, 8]. Cianfarani et al. discovered higher TSH concentrations in SGA children with blunted CUG, suggesting the intrauterine reprogramming may involve thyroid function, which might affect postnatal growth in turn [9].

Besides higher TSH concentrations, preterm SGA newborns are more susceptible to thyroid dysfunction, such as transient hypothyroidism and delayed TSH rise, due to the premature hypothalamic-pituitary-thyroid axis. Uchiyama et al. showed being SGA is a risk factor for the development of transient hypothyroxinemia with delayed TSH elevation (dTSHe) in CH in infants weighing less than 2000 g [10]. Furthermore, Kaluarachchi et al. found the percentage of SGA infants is significantly higher in the CH with dTSHe group [11].

Although the thyroid function in preterm SGA infants warrants further study, few publications addressed the altered thyroid hormone levels in the first week of life in this population. Therefore, we conducted the present study to verify the hypothesis that preterm SGA infants have higher TSH concentrations within the normal range and an increased incidence of thyroid dysfunction.

## Methods

### Study population and study design

The study population was preterm newborns (GA < 37 wk), including both SGA and AGA ones. SGA was defined as a birth weight below the 10th percentile for a given GA and sex. The retrospective single-center study was designed to compare thyroid hormone levels within the normal range and the incidence of thyroid dysfunction between the SGA and AGA groups to verify our hypothesis.

The medical records of all preterm infants admitted to the neonatal intensive care unit (NICU) at the First Affiliated Hospital of Shantou University Medical College, Shantou, China, between January 1, 2015 and December 31, 2018, were reviewed. Records were identified by the following ICD-9 codes: preterm, premature and small for gestational age. The exclusion criteria were: admission after 1 week of age, death, loss to follow-up, sepsis or other severe infectious diseases, maternal thyroid diseases and unavailable or incomplete records. After exclusion, 850 preterm infants (63 SGA and 787 AGA infants) entered into the final analysis.

The initial TFT was performed between 72 and 96 h of life after admission. The blood samples were drawn into serum separating tubes and stored at − 20 °C, then analyzed with TSH, FT4 and FT3 assays using the ADVIA Centaur Automated analyzer (Siemens Healthcare Diagnostics, Munich, Germany) on the same day.

The screening, diagnosis, and management of CH in the target population were performed strictly according to our institution protocol, which is in line with the Chinese and European consensus guidelines on screening, diagnosis, and treatment of CH [3, 12]. The normal range references of hormone levels are presented as following: TSH (1.3 ~ 9.91 mU/L for male, 0.77 ~ 19.42 mU/L for female), FT4 (11.85 ~ 33.81 pmol/L), and FT3 (2.63 ~ 5.70 pmol/L) [3, 12]. If the thyroid hormone levels were abnormal or L-T4 therapy started, more TFTs would be carried out to monitor until they were normalized.

The ethical committee of the First Affiliated Hospital of Shantou university medical college approved the study with a waiver of consent.

### Data collection and definition

Neonatal demographic and outcome data were extracted from the clinical database. The demographic characteristics included sex, BW and BW groups, GA and GA groups, being a twin, Caesarean section delivery, and in vitro fertilization. Secondary parameters, such as Z-score of BW for GA and sex, and Ponderal index (PI, defined as weight (g)/length (cm)^3^ × 100), were calculated.

The history of conditions such as 1- and 5-min Apgar scores, presence of respiratory distress syndrome, intraventricular hemorrhage, necrotizing enterocolitis, and cardiac problems, and NICU procedures such as respiratory support (invasive or noninvasive), surfactant administration, and use of medications (steroids, dopamine and furosemide), were collected.

The time points and modality of TFTs, and the treatment and follow-up information for each patient were extracted and categorized. The definitions of thyroid dysfunction were mainly based on the initial TFT result, although one set of TFTs were probably performed for the purpose of diagnosis and follow-up.

CH was defined as TSH > 40 mU/L in the initial TFT. The subjects with a definite diagnosis of CH were excluded from the analysis. Transient hypothyroidism was defined as FT4 <  11.85 pmol/L in conjunction with TSH ≥10 mU/L. Transient hypothyroxinemia (TH) was defined as FT4 <  11.85 pmol/L with TSH < 10 mU/L. dTSHe was defined as TSH > 20 mU/L following a normal result in the initial TFT. Hyperthyrotropinemia was defined as FT4 ≥ 11.85 pmol/L in conjunction with TSH ≥10 mU/L. Low T3 syndrome was defined as FT3 < 2.63 pmol/L, while FT4 and TSH levels were normal.

For the initial TFT results not defined above, a follow-up TFT was carried out in 2 weeks to decide whether LT-4 therapy was needed. According to our NICU protocol, if venous FT4 concentration was below norms for age, L-T4 treatment was started immediately. If venous TSH concentration was > 20 mU/L, treatment was started, even if FT4 concentration was normal. The first follow-up examination was performed within 2 weeks after the start of treatment, initiating intense follow-up with periodical TFTs over the first year of life until TSH levels were completely normalized.

### Statistical analysis

Statistical analysis was performed by using Stata version 12 (Stata Corporation, College Station, TX, USA). Normality test were applied to determine the data distribution. Continuous variables were expressed as the mean ± SD. or median with interquartile range if the data were skewed, and were compared using Student’s t-test or Wilcoxon rank-sum tests according to the data distribution. Categorical variables were reported as the number with percentage, and were compared using the chi-square test.

Stepwise linear multivariate regression was performed to identify risk factors of altered TSH levels with correction for potential confounders, including the demographic and clinical factors. Model 1 and 2 used TSH levels as the dependent variable, and the following factors as the independent variables:

- Demographic factors: being SGA or Z-score, sex, Ponderal index, being a twin, Caesarean section, and in vitro fertilization.
- Clinical conditions: low 1- and 5-min Apgar score, and presence of respiratory distress syndrome, severe IVH, necrotizing enterocolitis, and cardiac problems.
- Procedures and medications: respiratory support, surfactant administration, and use of steroids, dopamine, and furosemide.

Model 1 used being SGA as an independent variable, while Model 2 used the Z-score of BW for GA and sex as the variable in place of being SGA. Considering the BW and GA highly correlate with the two variables, we did not include them in the models. *P-*value significance was set at < 0.05. The two models were also used to examine the risk factors of altered FT4 levels.

## Results

Table 1 summarizes the demographic and clinical characteristics of the study population. In demographic factors, univariate conditional logistic regression shows that BW, GA, Z-score of BW for GA and sex, Ponderal index, and Caesarean section delivery are significantly different between the SGA and AGA groups. Among clinical factors, a higher percentage of low 1-min Apgar score (< 7) can be seen in the SGA group.

**Table 1:** Demographic and clinical characteristics of the SGA and AGA groups

	SGA	AGA	***P***-value
**Demographic factors**			
Participants, n	189	661	
Male sex	111 (58.73)	389 (58.85)	0.976
BW, kg	1.56 ± 0.37^*^	2.07 ± 0.51^*^	< 0.001
	1.58 (0.61 ~ 2.30)^**^	2.10 (0.86 ~ 5.07)^**^	
ELBW^#1^ (< 1 kg)	12 (6.35)	5 (0.76)	
VLBW^#2^ (1 ~ 1.5 kg)	68 (35.90)	96 (14.55)	
LBW^#3^ (> 1.5 kg ~ 2.5 kg)	109 (57.67)	447 (67.73)	
NBW^#4^ (> 2.5 kg)	0	112 (16.97)	
GA, wk. (week)	34.03 ± 2.05^*^	33.53 ± 2.39^*^	0.009
	35 (28 ~ 36.86)^**^	34.00 (26 ~ 36.86)^**^	
Extremely preterm (< 28wk)	2 (1.06)	19 (2.87)	
Early preterm (28 ~ 31^6/7^wk)	26 (13.76)	134 (20.27)	
Mild preterm (32 ~ 36^6/7^wk)	153 (85.19)	508 (76.85)	
Z-score	−1.85 ± 0.59	−0.14 ± 0.72	< 0.001
Ponderal index	2.29 ± 0.42	2.46 ± 0.27	< 0.001
Being a twin	59 (31.22)	154 (23.30)	0.027
Caesarean section	58 (30.69)	307 (46.59)	< 0.001
In vitro fertilization	20 (10.58)	83 (12.59)	0.455
**Clinical factors**			
Low Apgar score at 1 min(< 7)	30 (16.39)	52 (8.01)	0.001
Low Apgar score at 5 min(< 7)	13 (6.91)	45 (6.85)	0.975
Respiratory distress syndrome	65 (34.39)	239 (36.16)	0.655
Severe IVH^##^ (grade 3 or 4)	4 (2.14)	19 (2.89)	0.577
Necrotizing Enterocolitis	13 (6.91)	36 (5.46)	0.452
Cardiac problems	35 (18.62)	101 (15.40)	0.290
Respiratory support			
Invasive	33 (17.46)	88 (13.31)	0.150
Noninvasive	60 (31.75)	195 (29.50)	0.553
Surfactant administration	50 (26.46)	197 (29.80)	0.371
Medications			
Steroids	5 (2.64)	13 (1.97)	0.560
Dopamine	12 (6.35)	26 (3.93)	0.150
Furosemide	2 (1.05)	5 (0.76)	0.686

^#1-4^*ELBW* Extremely low birth weight; *VLBW* Very low birth weight; *LBW* Low birth weight; *NBW* Normal birth weight

^*^ Presented as mean ± standard deviation

^**^ Presented as median (range)

^##^*IVH* Intraventricular hemorrhage

Table 2 presents the following results: (1) thyroid hormone levels within the normal range, (2) the incidence and type of thyroid dysfunction determined by the initial or following TFTs, and (3) L-T4 administration and follow-up information.

**Table 2:** Thyroid hormonal levels within the normal range, incidences of thyroid dysfunction, and rates of treatment and follow-up in the SGA and AGA groups

	SGA(***n*** = 189)	AGA(***n*** = 661)	***P***-value
**Thyroid hormone levels within the normal range**^c^			
TSH (mU/L)^a^	3.74 (2.28 ~ 6.18)	3.01 (1.81 ~ 5.41)	0.018
FT4 (pmol/L)^b^	17.76 ± 3.94	17.42 ± 3.71	0.371
FT3 (pmol/L)	3.57 ± 0.73	3.51 ± 0.69	0.362
**Thyroid dysfunction determined by TFTs**			
Transient Hypothyroidism	4 (2.12)	10 (1.51)	0.565
Transient Hypothyroxinemia	22 (11.64)	83 (12.56)	0.576
TH with dTSHe^d^	3 (1.59)	2 (0.30)	0.005
Hyperthyrotropinemia	21 (11.11)	59 (8.93)	0.331
Low T3 syndrome	9 (4.76)	37 (5.60)	0.413
**Treatment and follow-up**			
L-T4 treatment	55 (29.10)	137 (20.73)	0.015
Follow-up months			
0 ~ 6 m	44 (23.28)	96 (14.75)	0.004
7 ~ 12 m	5 (2.65)	16 (2.46)	0.861
13 ~ 18 m	1 (0.53)	4 (0.61)	0.904
> 18 m	4 (2.12)	9 (1.38)	0.456

^a^ Presented as median (interquartile range)

^b^ Presented as mean ± standard deviation

^c^ Normal range: TSH 1.3 ~ 9.91 mU/L for male, 0.77 ~ 19.42 mU/L for female; FT4 11.85 ~ 33.81 pmol/L; FT3 2.63 ~ 5.70 pmol/L

^d^ TH with dTSHe: Transient hypothyroxinemia with delayed TSH elevation

TSH levels are significantly higher in the SGA group than the AGA group ((3.74(interquartile range (IQR): 2.28 ~ 6.18) vs. 3.01(IQR:1.81 ~ 5.41) mU/L, *p* = 0.018). The incidence of TH with dTSHe is significantly higher in the SGA group (1.59% vs. 0.30%, *p* = 0.005). The SGA infants have a higher rate receiving L-T4 therapy (29.10% vs. 20.73%, *p* = 0.015), and a higher follow-up percentage in the first 6 months of life (23.28% vs 14.75%, *p* = 0.004).

Table 3 reports the result of stepwise multivariate regression by using two models. Model 1 used SGA birth as a categorical variable, and Model 2 used Z-score of BW for GA and sex as a quantitative surrogate of SGA birth. TSH levels remained significantly associated with SGA birth (0.68(95%CI 0.15 ~ 1.21), *p* = 0.013), or Z-score (− 0.25 (95%CI -0.48 ~ − 0.03), *p* = 0.028) after adjusting for potential confounders. In other words, Being SGA accounts for 0.68 mU/L TSH elevation, or 1 unit Z-score decrease is related to 0.25 mU/L TSH elevation. We further stratified the study population based on GA and BW groups, then run the regression models in each group. The results showed the associations remained significant in very low birth weight group (BW 1 ~ 1.5 kg) and mild preterm (GA 32 ~ 36^6/7^wk) group, respectively.

**Table 3:** Risk factors of TSH elevation within the normal range in the study population

Variables	***β***coefficient	95% CI	***P***-value
**Model 1**			
SGA birth	0.68	0.15 ~ 1.21	0.013
Necrotizing enterocolitis	1.05	0.05 ~ 2.04	0.039
**Model 2**			
Z-score	−0.25	−0.48 ~ −0.03	0.028
Necrotizing enterocolitis	1.06	0.07 ~ 2.05	0.036

As FT4 concentrations were found significantly higher in the SGA group in the univariate analysis, we also applied the two models to examine the risk factors of altered FT4 levels in the study population. However, we did not find a significant association between SGA birth and altered FT4 concentrations.

## Discussion

The present study found that TSH levels were significantly higher in SGA newborns than AGA ones. The association between higher TSH levels and SGA birth was further confirmed by the following multivariate regression analysis after adjusting for potential confounders, including the history of conditions, procedures, and medications before the initial TFT.

Taking advantage of the TFT results, our discovery have confirmed and expanded the findings of previous studies. Bosch-Giménez et al. reported higher TSH concentrations in SGA neonates, but their data were from NBS which measured TSH exclusively, and the TSH level range was set to < 7.5 mU/L [6]. In our study, thyroid hormone values were from those infants with normal thyroid function diagnosed by one or more TFTs, hence our TSH values were higher than theirs. Franco et al. reported that TSH concentrations are significantly higher only in term SGA infants compared to AGA ones, whereas serum concentrations of T4 are lower in both preterm and term SGA infants [13]. FT4 concentrations were significantly higher in the SGA group, although the significance did not hold in multivariate linear regression later. Nonetheless, our conclusions are in line with the two studies that SGA babies have a higher incidence of transient hypothyroidism and need close follow-up.

In the present study, the higher percentages of low 1-min Apgar score and Caesarian section delivery in the SGA group were seen. SGA infants do not have relatively mature functions of organ systems that AGA infants possess, so they are more susceptible to birth asphyxia and difficult deliveries, which tend to elevate TSH levels. Lower Apgar score, known as an indicator of asphyxia at delivery, has been associated with higher TSH levels [14, 15]. However, Rashmi et al. found the lowest TSH levels in infants born by elective Caesarian section delivery compared to other modes of delivery, suggesting it may be a factor decreasing TSH levels [15].

Besides altered thyroid hormone concentrations, we presented different kinds of thyroid dysfunction diagnosed by one or more TFTs in Table 2. The SGA infants showed a significantly higher incidence of transient hypothyroidism with dTSHe. Recently, a Japanese study showed that SGA birth is the only independent risk factor for the development of TH with dTSHe in the preterm infants weighing less than 2000 g [10]. Another study revealed that the prevalence of CH with dTSHe is associated with SGA birth in extremely and early premature infants (GA <30wk) [11]. Our finding further underlines preterm SGA infants are prone to this disorder and should be closely followed up.

Additionally, we presented the rates of starting on L-T4 replacement therapy and follow-up in the SGA and AGA groups. The rate of SGA infants receiving the therapy was significantly higher than that of AGA ones, indicating the SGA infants suffered more from a deranged thyroid hormone secretion. The follow-up rate within the first 6 months of life was significantly higher in the SGA group, but the significance did not persist beyond. Compared to the AGA group, the SGA group was more likely to have thyroid dysfunction and longer treatment, but in most cases, the alterations were transient and reverted to normal during the following months. The incidence affected by hypothyroidism in the two groups did not show a significant difference in the long term.

Several lines of research addressed TSH elevation in fetuses and children born SGA. Thorpe-beeston et al. found that significantly higher levels of TSH and lower levels of T4 and FT4 in SGA fetuses, and suggested the associations may be explained by degrees of fetal hypoxemia and academia respectively [16]. The authors also revealed some SGA fetuses may have abnormal thyroid function.

Radetti et al. found that TSH concentrations are significantly higher in children born SGA, and 20% SGA children have TSH levels above the upper limit of the normal range, whereas no difference was found for FT4 [17]. De Kort et al. found higher TSH levels within the normal range in preterm short SGA children, but mean FT4 is not significantly different [18]. Although these findings cannot lend direct support to our conclusion, they distinctly suggest that TSH elevation may play a similar role at different stages of the developmental process.

As thyroid hormones have been credited with a wide range of important physiologic functions, the etiology of higher TSH levels in SGA infants is worth exploring. It is postulated that many factors may involve, including immaturity of the hypothalamic-pituitary-thyroid axis, uterine stress with growth restriction, less efficient thermogenic response, or non-thyroidal illness. However, it is difficult to figure out the temporal pattern of thyroid hormone alteration caused by SGA stunting and to distinguish it from that of AGA infants, as either NBS or TFT is generally done at separate time points. Furthermore, exposure to various medications in hospitalization and the inability to regulate iodine balance may be other common reasons [19].

Given the importance of proper thyroid function, especially for the overall development in early and later childhood, some cohort studies investigated the impact of high neonatal TSH levels on long-term developmental and cognitive sequelae.

Differential effects of preterm and SGA birth on cognitive and motor development have been noted: SGA birth is associated with cognitive ability, as measured by IQ and reading comprehension, while motor ability was additionally associated with preterm birth [20]. Furthermore, Trumpff et al. reported lower verbal IQ scores in preschool children with high TSH values between 10 and 15 mU/L in univariate analysis, but the result did not hold after adjusting for confounding factors [21]. Chung et al. confirmed preterm infants with persistently high TSH levels have worse neurological outcomes compared to those with transiently high TSH levels [22]. Nonetheless, inconclusive results demonstrate the clinical significance of neonatal TSH elevation is still under debate [23, 24]. Considering the potential cognitive risks in infancy and childhood, preterm SGA infants with TSH elevation should be closely followed up.

The main strength of the present study is that we both evaluated the normal thyroid hormone concentrations and thyroid dysfunction in the study population.

First, the Z-score of BW for GA and sex was applied to quantify the association of altered thyroid hormone levels and SGA birth. Most previous studies interpret altered thyroid hormone levels and thyroid function from the perspective of prematurity, and analyze the data based on either GA or BW. The present study elucidated the thyroid alterations and disorders from the perspective of BW adequacy for GA.

Second, the primary and following TFTs enabled us to fully interpret the status of thyroid hormone secretion and thyroid disorders in the preterm newborns. Serum TFT measures TSH and FT4 simultaneously, which is considered the ideal screening approach to investigate the thyroid function. Evidence indicates that the screening based on measuring only TSH levels cannot diagnose transient hypothyroidism, so its clinical significance is fairly limited [25]. Therefore, measuring both FT4 and TSH in screening tests is advisable.

Third, a detailed collection of clinical risk factors facilitated the adjustment for these potential confounders in the multivariate regression analysis. As our study population was preterm SGA and AGA neonates admitted into NICU, the clinical confounding effects might show false associations if we failed to control them.

The present study has several limitations. First, the number of cases is relatively small compared to large-scale NBS, and further studies including more cases are warranted to confirm the findings. Second, it is a retrospective, single-center study in nature, and the data were extracted from the NICU database, thus our results may not be generalizable to other populations. Third, as the study subjects were hospitalized in the NICU, some confounding factors such as illness severity, procedures, and medications were inevitably introduced [26]. Although we have applied multivariate regression analysis to adjust for these potential confounders, bias and misclassification may not be ruled out, so the results should be interpreted cautiously.

## Conclusions

In conclusion, preterm SGA newborns had significantly higher TSH concentrations within the normal range and an increased incidence of thyroid dysfunction, but most of them were transient and reverted to normal after L-T4 correction within 6 months of life. SGA newborns with these features should be closely followed up with periodical TFTs and endocrinologic evaluation.

## Data Availability

The datasets used and/or analyzed during the current study are available from the corresponding author on reasonable request.

## Abbreviations

SGA: Small for gestational age
AGA: Apropriate for gestational age
GA: Gestational age
NBS: Newborn screening
TFT: Thyroid function test
TSH: Thyroid stimulating hormone
FT4: Free thyroxine
FT3: Free tri-iodothyroxine
dTSHe: Delayed thyroid stimulating hormone elevation
TH: Transient hypothyroxinemia
ELBW: Extremely low birth weight
VLBW: Very low birth weight
LBW: Low birth weight
NBW: Normal birth weight
L-T4: levothyroxine
IVH: Intraventricular hemorrhage
NICU: Neonatal intensive care unit
OR: Odds ratio
CI: Confidence interval
IQR: Interquartile range
